# Immunomodulatory effects of excretory/secretory compounds from *Contracaecum osculatum* larvae in a zebrafish inflammation model

**DOI:** 10.1371/journal.pone.0181277

**Published:** 2017-07-24

**Authors:** Foojan Mehrdana, Per Walter Kania, Sasan Nazemi, Kurt Buchmann

**Affiliations:** 1 Laboratory of Aquatic Pathobiology, Department of Veterinary and Animal Sciences, Faculty of Health and Medical Sciences, University of Copenhagen, Frederiksberg C, Denmark; 2 Section of Experimental Animal Models, Department of Veterinary and Animal Sciences, Faculty of Health and Medical Sciences, University of Copenhagen, Frederiksberg C, Denmark; National Center for Toxicological Research, UNITED STATES

## Abstract

Excretory/secretory (ES) compounds isolated from third-stage larvae of the anisakid nematode *Contracaecum osculatum* parasitizing liver of Baltic cod were investigated for effects on immune gene expression in a zebrafish LPS-induced inflammation model. ES products containing a series of proteins, of which some had enzymatic activity, were injected solely or with LPS. ES proteins alone induced up-regulation of a number of immune-related genes, but generally to a lower degree compared to LPS. When co-injected with LPS, the worm products exacerbated merely expression of five genes affecting Th1, Th2, Th17 and innate responses compared to the LPS-injected group. However, the level of overexpression decreased in an inverse dose-dependent manner. The immune regulating action of *C*. *osculatum* ES products is interpreted as an important evolutionary ability of larval parasites in the transport host which makes it less susceptible to host immune responses whereby the probability of reaching the final host is increased.

## Introduction

Parasitic helminths produce a series of excretory/secretory (ES) compounds which have been suggested to play an important role in parasite-host interactions [1]. In nematodes this complex of molecules originates from different organs of the parasite (oesophagus, ventricle, intestine, glands) and comprises various enzymes with different functions in the host including penetration and migration in the host tissues, alteration of host physiology, and immunomodulation with the purpose of favouring parasite survival [1, 2]. Enzymes such as acetylcholinesterase (AChE), glutathione-S-transferase (GST), and superoxide dismutase (SOD) secreted by the hookworm *Necator americanus* act as anti-inflammatory molecules creating a shielded pathway in order to protect the worm from immune reactions [3]. Correspondingly, the filarial nematode *Wuchereria bancrofti* produces AChE in the human host circulation which degrades acetylcholine, inhibits lysosomal enzyme release and phagocytosis [4]. ES compounds have been suggested as potential therapeutics for inflammatory disorders. Thus, in a murine experimental asthma model it was shown that the *Ascaris suum* ES protein (PAS-1) is able to suppress allergen-induced Th2 responses, inhibit cellular migration, suppress cytokine expression (IL-4, IL-5), and reduce chemokine production in bronchoalveolar tissues [5]. We, therefore, hypothesize that ES products of third-stage nematode larvae of *Contracaecum osculatum* possess similar immunoregulatory properties. The life cycle of *C*. *osculatum* (Rudolphi, 1802) comprises adult worms in marine mammals, e.g. seals [6, 7], and infective third-stage larvae in invertebrates and teleosts serving as intermediate/transport hosts. Humans may accidentally obtain third-stage larvae of the parasite through consumption of raw or under-processed seafood which causes anisakidosis associated with gastrointestinal symptoms [8–10] and experimental *C*. *osculatum* infections of pigs elicit eosinophilic granuloma formation [11]. Other ascarid nematodes, e.g. *Ascaris lumbricoides*, *Toxocara canis*, and *Anisakis* spp. [12–16] produce a series of immunogenic molecules, including allergens, which suggests that the immunogenicity of *C*. *osculatum* proteins should be addressed. The occurrence of *C*. *osculatum* is increasing in certain localities, e.g. the Baltic Sea [7, 17] and may have an increasing influence on health in fish and mammals including humans. It is, therefore, worthwhile to investigate the immunogenic properties of *C*. *osculatum*, and we here present data on immunoregulation by ES proteins from this parasite elucidated in a zebrafish inflammation model. This experimental fish represents some advantages (small size, ease of handling and breeding, and rapid life cycle) compared to rodent models [18]. Therefore, zebrafish is currently being applied in biomedical research including immunology, as innate and adaptive immune responses are highly evolutionarily conserved. Thus, both fish and mammals share similar sets of immune signalling molecules and immune cells (e.g. cytokines, neutrophils, macrophages, dendritic cells, B and T cells) [19] and zebrafish models have been used in the studies of human inflammatory disorders such as hepatic inflammation and inflammatory bowel disease (IBD) [20, 21]. To date, no animal model has directly used ES compounds from *C*. *osculatum* for immunomodulation. Thus, in the present study we applied a lipopolysaccharide (LPS)-induced inflammation model in zebrafish aiming to elucidate whether *C*. *osculatum* ES compounds have any effects on the inflammatory responses.

## Materials and methods

### Ethics statement

The experimental protocol was approved by the Experimental Animal Inspectorate under the Ministry of Food, Agriculture and Fisheries (license no: 2016-15-0201-00902). No animals died prior to the experimental endpoint and etomidate was used during anaesthesia and euthanization.

### Parasites

Third-stage larvae of the anisakid nematode *C*. *osculatum* were isolated from livers of 34 specimens of Atlantic cod (Baltic subpopulation of *Gadus morhua)* caught by a local fisherman in ICES SD 25, east of the island Bornholm, Southwestern Baltic Sea. Infected livers were transferred to poly-ethylene bags immediately after catch and kept on ice during transport to the laboratory. Livers were then incubated in a pepsin/HCl/NaCl solution with magnetic stirring (250 rpm) at 37°C [22] using a volume of 10 ml pepsin solution per gram fish liver. Following full digestion of livers (1–2 h), the digest was filtered through a 300 μm sieve and the isolated nematodes were collected.

### ES product isolation

Recovered parasites were subsequently washed several times in phosphate buffered saline (PBS) and then incubated at 37°C in sterile 12-well Nunclon^TM^ cell culture plates (WVR, Denmark) for five days. Each well contained 10 live larvae in 2.5 mL PBS with antibiotics (200 μg/mL ampicillin and 400 μg/mL kanamycin sulphate) (Sigma-Aldrich, Denmark). Dying larvae were removed on a daily basis to avoid contamination with somatic proteins. After incubation the nematodes were removed and the media was filtered through 0.20 μm Minisart^®^ filters (Sigma-Aldrich, Denmark) and stored at -40°C until further use. Subsamples from wells were inoculated on blood agar plates and kept at 37°C for 48 h to confirm the lack of any bacterial growth in the media. In order to concentrate (2085 μg protein/mL measured by Nanodrop 2000 spectrophotometer, Saveen & Werner ApS, Denmark) and desalt ES solutions and remove antibiotics, we used Centriprep^®^ centrifugal filters (Cut-off value 3000 Da) with Ultracel-3 membrane (Merck Millipore, Denmark) according to the manufacturer´s instructions.

### SDS-PAGE

The ES products were subjected to SDS-PAGE to assess the size of the purified proteins. The sample was diluted in NuPAGE^®^ LDS Sample Buffer (4X) and NuPAGE^®^ Reducing Agent (10X) according to the manufacturer´s instructions (Invitrogen, Denmark) and boiled at 70°C for 10 min. It was subsequently applied on pre-casted NuPAGE gels (4–12% NuPAGE Bis-Tris gels, Invitrogen, Denmark) using NuPAGE MES SDS running buffer in a XCell SureLock^TM^ electrophoresis cell (Invitrogen, Denmark) at 200 V for 45 min whereafter protein bands were visualized by silver staining.

### Enzyme activity assay of ES proteins

In order to determine enzymatic activity of *C*. *osculatum* ES proteins, the API^®^ ZYM system (Biomerieux SA, Sweden) was applied. This is a semi-quantitative method testing 19 enzyme reactivities. The system consists of 20 cupules containing enzymatic substrate in the base and any enzymatic reactivities are detected through coloured reactions rated according to a table provided by the manufacturer. ES concentrate (65 μL of ES to each well) was incubated for 4 h and 40 min at 37°C whereafter reagents ZYM A and ZYM B were added and allowed to react for 5 min for colour development.

### Inflammation model

We used a total number of 80 adult AB wild-type zebrafish, age 11 months [mean body weight of 387.8 (SD: 104.4) mg and mean body length of 29.9 (SD: 2.28) mm] which were provided by the Panum Institute, University of Copenhagen and reared in a thermostat-controlled room at 28°C with a 12 h light: 12 h dark cycle. The fish were divided into eight experimental groups each containing 10 fish in duplicate (5 fish/tank); PBS-injected group (control), LPS-injected group using phenol-purified LPS from *E*. *coli* 0111:B4 (L2630, Sigma) (1.5 mg/mL), three ES-injected groups treated with different concentrations (low: 50 μg/mL, medium: 500 μg/mL, high: 1000 μg/mL), and three LPS+ES-injected groups treated with different concentrations of ES as explained earlier.

Fish were anesthetized by immersion into a solution of 2 mg/L etomidate (Sigma-Aldrich, Denmark). In each group, individual fish were injected intraperitoneally (i.p.) with a total volume of 20 μL solution. Intraperitoneal injection was performed using a Biohit automatic pipette (Dandiag, Denmark) mounted with a sterile BD Microlance^TM^ 3 needle (BD, Denmark) on a modified 200 μL pipette tip (Almeco A/S, Denmark) under a stereomicroscope (Leica MZ12.5, Leica Denmark). After injection, fish were returned to their tanks and observed every 2 hours in order to remove and euthanize any moribund fish from the tanks.

### Sampling

Twenty four hours after injection, the fish were euthanized with an overdose of etomidate solution (30 mg/L). Viscera including intestine, liver, and spleen were removed with fine forceps and immediately transferred to RNAlater^®^ (Sigma-Aldrich, Denmark) and kept at +4°C for 24 h until processing for gene expression studies.

### RNA purification and cDNA synthesis

Visceral tissues were lysed for 2 min at a frequency of 20Hz in a tissue-lyser II (Qiagen, Denmark) in 300 μL lysis buffer containing 2-mercaptoethanol (Sigma-Aldrich, Denmark). Total RNA was extracted using the GenElute^TM^ mammalian total RNA kit (Sigma-Aldrich, Denmark) according to the manufacturer´s instructions. Genomic DNA contamination was removed by DNase I (ThermoFisher Scientific, Denmark) treatment and RNA concentration was measured using NanoDrop 2000 spectrophotometer (Saveen & Werner ApS, Denmark). RNA purity and integrity was assessed using 1.5% ethidium bromide-stained agarose gel electrophoresis (Invitrogen, Denmark). The RNA was stored at -80°C.

Synthesis of cDNA was performed in a Biometra T3 thermocycler (Fisher Scientific, Germany) using a total of 100 ng of RNA with Oligo d(T)16 primer and MultiScribe^TM^ reverse transcription reagents (Applied Biosystems, Denmark) in a 20 μL of reaction volume. Reaction conditions were 25°C for 10 min, 37°C for 60 min, and 95°C for 5 min. The cDNA was diluted 10 times into 200 μL with RNase-free water (Invitrogen, Denmark) and stored at -20°C.

### Real-time quantitative polymerase chain reaction (RT-qPCR)

RT-qPCR analysis was conducted in an AriaMx Real-Time PCR machine (AH diagnostics A/S, Denmark) using a panel of probe-based assays [23]. The 12.5 μL reaction volume was composed of 2.5 μL cDNA, 6.25 μL Brilliant II QPCR Master Mix (AH diagnostics A/S, Denmark), 0.5 μL of a mix of forward primer (10 μM), reverse primer (10 μM), and probe (5 μM), and 3.25 μL RNase-free water. Cycling conditions was 95°C for 15 min followed by 40 cycles of denaturation at 95°C for 10 sec and combined annealing/elongation step at 60°C for 45 sec. The investigated genes encoded cytokines [IL-1β, IL-4/13, IL-6, IL-8, IL-10, IL-12, IL-17, IL-22, IL-23, IFNγ, TGFβ, TNFα], acute phase proteins [SAA, CRP, C3], toll-like receptors and adaptors [TLR2, TLR3, Ticam1, Myd88], cellular receptors [CD4, CD8], immunoglobulins [IgM, IgZ], matrix metalloproteinase-9 [Mmp9], transcription factors [FoxP3, GATA3, NFκB, STAT3, STAT4, STAT6,T-bet], and S100A1. Reference genes applied were *EF-1α*, *RPL13*, and *β-actin*. Sequences of primers and probes are shown in S1 Table.

### Data analysis

Duplicate groups were combined as they did not differ significantly. The differences in gene expression levels between different treatment groups were compared using student’s t-test. Data are presented as mean expression of 10 individual zebrafish as fold change ± standard error (fold ± SE). Difference in expression levels is considered significant at a probability level of 5% and fold changes of at least 2.

## Results

### SDS-PAGE

**Fig 1:**
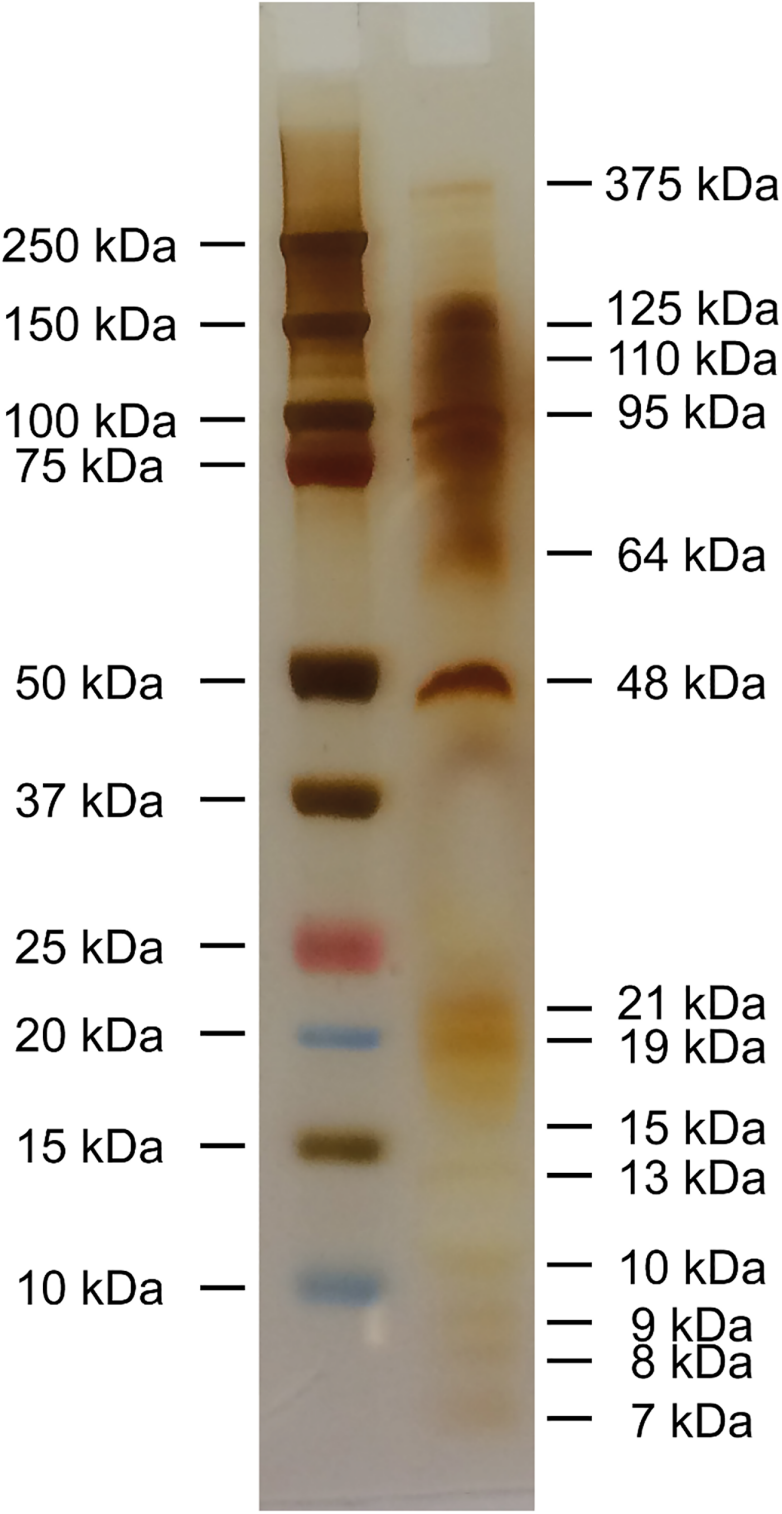
SDS-PAGE of the isolated ES proteins from *C*. *osculatum* third-stage larvae; reducing condition, silver staining; Left column: Marker Precision Plus Protein^TM^ Dual Color Standards; Right column: *C*. *osculatum* ES proteins with different molecular weights.

Silver staining after SDS-PAGE revealed a series of protein bands with molecular weights (MW) ranging from 7 kDa to 375 kDa. Dominating bands comprised, with falling intensities, proteins with MWs at 48, 95, 125, and 110 kDa (Fig 1).

### Enzyme activity of ES proteins

High enzyme activity of the ES protein solution was recorded with regard to acid phosphatase and naphthol-AS-BI-phosphohydrolase, followed by esterase, leucine arylamidase, and α-glucosidase showing medium activity. Alkaline phosphatase, esterase lipase, and N-acetyl-ß-glucosaminidase showed low activity. No activity was recorded for the other enzymes as well as control (Table 1).

**Table 1:** Enzyme activity of excretory/secretory proteins produced by *C*. *osculatum* third-stage larvae.

Enzymes	ES protein	PBS
Alkaline phosphatase	1	0
Esterase (C 4)	3	0
Esterase Lipase (C 8)	1	0
Lipase (C 14)	0	0
Leucine arylamidase	3	0
Valine arylamidase	0	0
Cystine arylamidase	0	0
Trypsin	0	0
α-chymotrypsin	0	0
Acid phosphatase	5	0
Naphthol-AS-BI-phosphohydrolase	5	0
α-galactosidase	0	0
ß-galactosidase	0	0
ß-glucuronidase	0	0
α-glucosidase	2	0
ß-glucosidase	0	0
N-acetyl-ß-glucosaminidase	1	0
α-mannosidase	0	0
α-fucosidase	0	0

Phosphate buffer saline (PBS) was used as negative control. According to the colours developed, a value ranging between 0 and 5 is assigned; 0 corresponds to a negative reaction and 5 to a reaction with maximum intensity.

### Gene expression

Adult wild-type zebrafish were i.p. injected with LPS and/or three different concentrations (low, medium, and high) of *Contracaecum* ES proteins and after 24 h fish were sampled for recording of immune genes expression using RT-qPCR. The survival rate in all examined groups was 100%.

The general expression level of each individual gene for all experimental groups indicated highest expression in *C3*, followed by *CRP*, *NFκB* and *S100A1* genes, while *IL-10*, *IL-22*, and *IL-12* had the lowest level of expression (S1 Fig).

**Fig 2:**
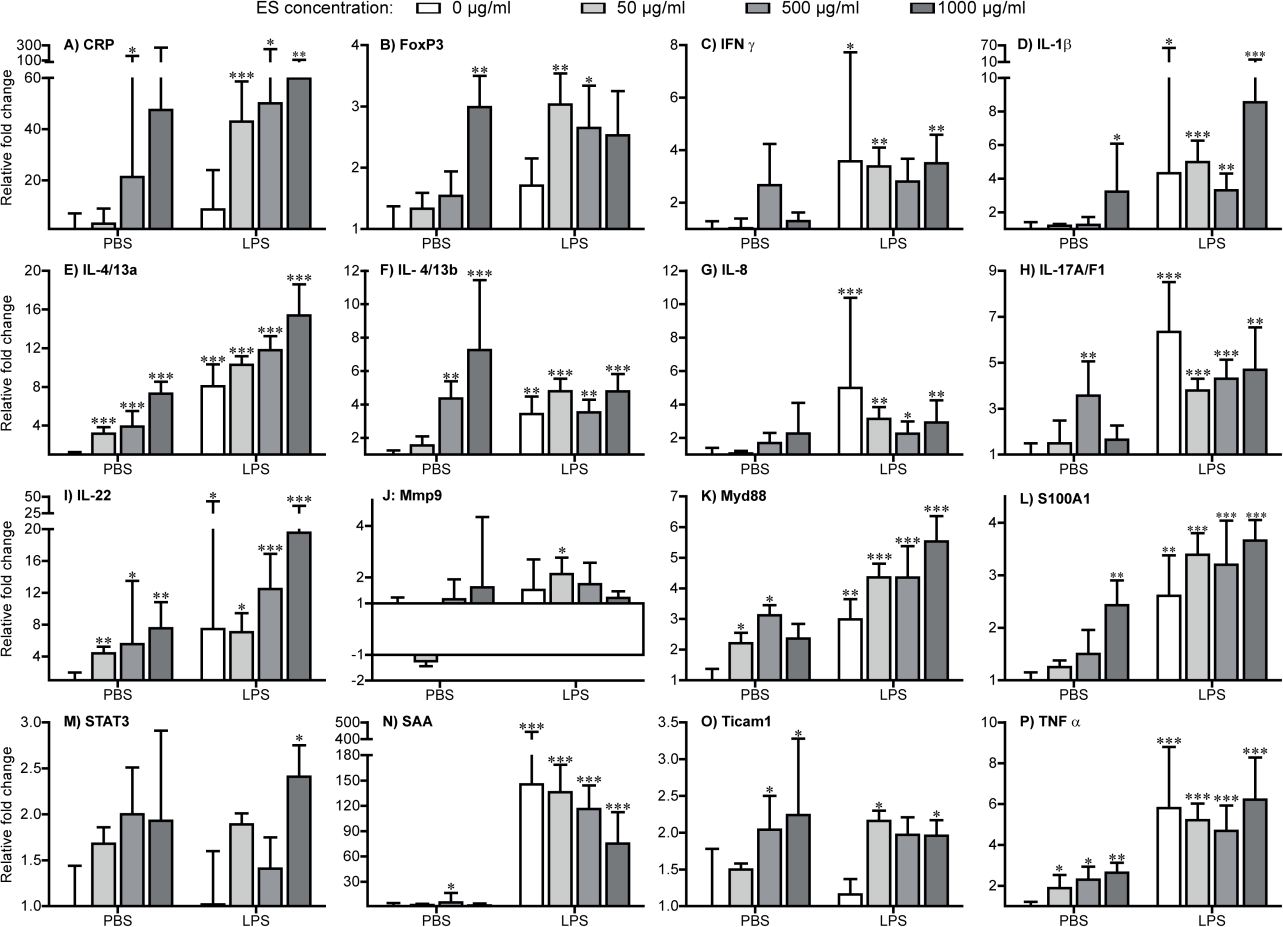
Expression of immune genes in zebrafish after exposure to PBS or LPS plus ES compounds. Fold change in all groups are compared to expression in PBS-injected zebrafish. As the efficiencies of all the assays were within 100% ± 5%, the simplified 2 –ΔΔCq method were used to calculate the relative fold change. Asterisk sign: significance level of fold changes of each gene (*P < 0.05; **P <0.01; ***P < 0.001) and fold changes of at least 2. The elongation factor 1α (*EF-1α*) was used as the reference gene.

**Fig 3:**
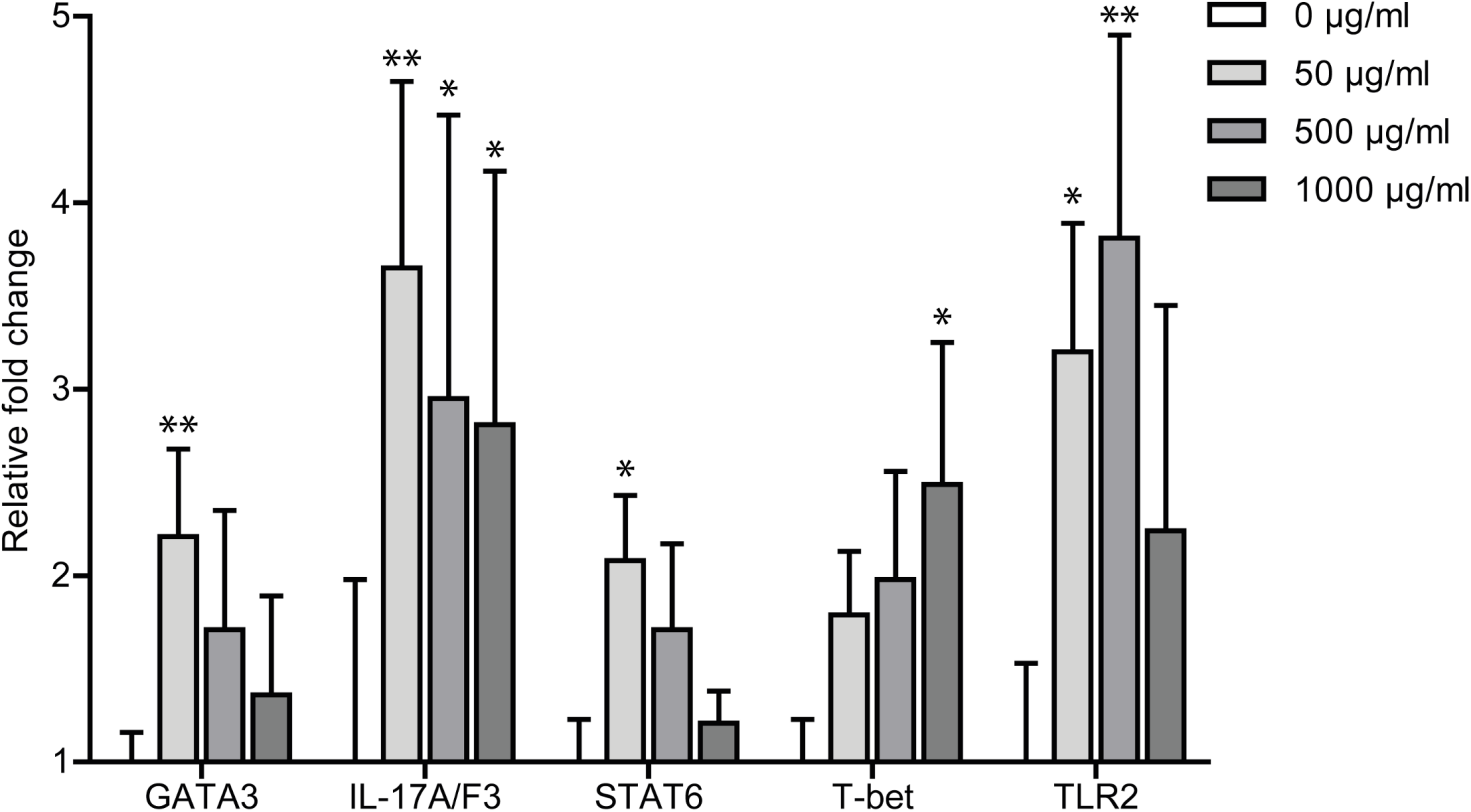
Expression of immune genes in zebrafish after exposure to LPS or LPS plus ES compounds. Fold change ± standard error (fold ± SE) are compared to expression level in LPS-exposed fish. Difference in expression levels is considered significant at P values smaller than 5% and fold changes of at least 2. Asterisk sign: significance of fold changes of each gene in LPS+ES groups compared to the LPS-only group (*P < 0.05; **P <0.01; ***P < 0.001).

1. **ES-treated fish compared to PBS control**.
  - Cytokines: A significant concentration-dependent up-regulation in expression of the IL-4/13a, IL-4/13b, IL-22, and TNFα genes was found after exposure to ES compounds. The genes encoding IL-1β and IL-17A/F1 were significantly up-regulated following exposure to high and medium concentrations of ES proteins, respectively (Fig 2). No significant regulatory changes were observed with regard to other cytokine genes.
  - Transcription factors: Only the high concentration of ES proteins induced significant up-regulation of the FoxP3 gene. No markedly altered expression level of STAT3 was revealed (Fig 2).
  - Other molecules: Transcripts of the gene encoding S100A1 was significantly up-regulated in the group treated with a high concentration of ES compounds. A slight up-regulation in expression of genes encoding CRP, SAA, Myd88, and Ticam1 was recorded (Fig 2).
2. **LPS-treated fish compared to PBS control.** All tested immune-related genes were significantly up-regulated following LPS injection with exception of genes encoding STAT3, Ticam1, FoxP3 and CRP (Fig 2).
3. **LPS+ES-treated fish compared to PBS control.** All genes presented in Fig 2 at all ES concentrations were significantly up-regulated following co-stimulation with LPS and ES compounds with the exception of Mmp9, which showed significant up-regulation only at the highest concentration of ES.
4. **LPS+ES-treated compared to LPS only-treated fish.** Co-stimulation of LPS-treated fish with ES compounds did only in few cases affect gene regulation significantly as compared to the LPS only-treated group. The expression of cytokine *IL-17A/F1* gene was significantly higher in the ES-treated fish, but with a falling trend with increasing ES concentration (Fig 3). Genes encoding the transcription factors GATA3 and STAT6 were distinctly up-regulated in fish co-stimulated with LPS and low concentrations of ES, whereas the *T-bet* gene showed a higher expression level following treatment with the high concentration of ES (Fig 3).

The *TLR2* gene was markedly up-regulated in the groups receiving LPS plus low and medium concentrations of ES proteins when compared to the LPS-only group (Fig 3).

Some examined genes were expressed at low levels, especially in the control group. Therefore, quantitative assessment was not possible. Thus, those genes are assessed only qualitatively in the S2 Table. In the case of *IL-6*, the groups receiving both LPS and ES had significantly higher number of valid Cq values as compared to the PBS control fish. These groups were defined as qualitatively up-regulated. Likewise, all LPS-treated groups exhibited *IL-12* qualitative up-regulation and *IL-17A/F2* and *IL-17A/F1* were qualitatively up-regulated except in the group receiving the lowest dose of ES.

## Discussion

The zebrafish, *Danio rerio*, was in this investigation applied as a model for inflammation and found useful for studies of inflammation regulatory molecules. This fish species showed to react strongly to injection of LPS and a long range of genes encoding inflammatory factors such as cytokines and acute phase reactants were subsequently up-regulated. We found that LPS triggered production of proinflammatory cytokines (IL-1β, IL-8, and TNFα) and modulated gene expression related to the Th2 (*IL-4/13a*, *IL-4/13b*) and Th17 (*IL-17A/F1*, *IL-22*) signalling pathways. A 3-fold increase in *Myd88* gene expression and an extreme up-regulation of the acute phase protein *SAA* gene was observed due to LPS exposure. Mammals apply TLR4 for LPS recognition, while most fish lack this receptor [24–26], but it has previously been shown that zebrafish respond to LPS stimuli and produce an inflammatory reaction even from an early stage of 2 days post fertilization, although the responsible receptor has not yet been identified [27].

The subsequent aim of this study was elucidation of the immune-regulating functions of ES compounds from the nematode *C*. *osculatum*. A range of parasitic nematodes have previously been shown to produce and release molecules with anti-inflammatory effects also across the species barrier. We detected activity of several enzymes in the ES isolate from *C*. *osculatum* suggesting that a range of immune-relevant molecules such as Igs and complement factors could be directly broken down or modified. The parasite *A*. *suum* is able to suppress a series of inflammatory reactions in an experimental model of asthma in mice [5], and infections with the pig whipworm *Trichuris suis* have even been suggested as a therapy against intestinal disorders (IBD) in humans [28]. In addition, released compounds from the hookworm *N*. *americanus* [3] and *W*. *bancrofti* [4] was found to suppress Th2 responses, inhibit cellular migration and suppress cytokine expression. It could, therefore, be hypothesized that anisakid nematodes such as the widely distributed *C*. *osculatum* in gadid fish [17, 29, 30] may produce similar molecules with corresponding functions. The SDS-PAGE analysis of *C*. *osculatum* ES compounds clearly demonstrated the composed nature of the isolates and it cannot be excluded that some of the proteins may have dual functions; i.e. some may be inflammatory and others may be regulatory. As we applied concentration method removing smaller molecules, it should be noted that this study presents the effects of ES proteins with molecular weight above 3000 Da. The applied techniques for studying expression of both innate and adaptive immune genes in zebrafish were recently developed [23, 31] and we demonstrated that ES products from *C*. *osculatum* activate most immune genes in a dose-dependent manner, but generally at a markedly lower level compared to LPS. It is noteworthy that a high concentration of *C*. *osculatum* ES proteins also induced higher expression of the *FoxP3* gene indicating activation of T regulatory cells (Tregs), which in turn produces inhibitory and anti-inflammatory cytokines. Thus, it was generally shown that not only LPS but also ES compounds in themselves up-regulate a series of immune genes when compared to control zebrafish only receiving PBS. When the gene expression level of LPS/ES co-injected zebrafish was recorded and compared to the fish only injected with LPS, a significant up-regulation of genes encoding GATA3, IL-17A/F3 and STAT6 was noted. However, the expression level of these genes had an inverse relationship with the concentration of injected ES proteins; i.e. higher doses of ES compounds led to lower expression of the genes. GATA3 and STAT6 molecules are Th2 transcription factors, and IL-17A/F3 is an inflammation-associated cytokine. So, our findings demonstrated simultaneous inflammatory and immune-dampening effects of ES compounds from *C*. *osculatum* in a dose-dependent manner. This corresponds to previous investigations of *A*. *suum*, *T*. *suis*, *W*. *bancrofti* and *N*. *americanus* suggesting that products from these nematodes actively modulate the host immune system in different directions.

The relevance of using zebrafish as a general model to study immunity is based on the fact that fish immune genes, in particular innate immune-related genes, have been highly conserved throughout evolution and have counterparts even in mammalian genomes [32]. In zebrafish a number of counterparts for TLR-signaling genes (*TLR1*, *TLR2*, *TLR3*, *TLR4*, *TLR5*, *TLR7*, *TLR8*, *TLR9*), genes encoding cytokines such as *IFNγ* [33], interleukin receptors and associated adaptor proteins like *MyD88* [34], and signal transducer and activator of transcription genes (*STAT2*, *STAT3*, *STAT4*, *STAT6*) [32] have been characterized. Further, a series of genes encoding central molecules in adaptive responses such as *IgM*, *IgZ*, *CD4*, *CD8* and *MHC* have been detected [23] and in this study we focused on the central genes.

As some of the immune regulating actions of ES products may rely on enzyme activity, we also investigated, in this work, if the *C*. *osculatum* ES proteins exhibit enzymatic activities. SDS-PAGE clearly revealed that the worm excretions comprise numerous proteins and subsequent enzyme assays demonstrated the presence of a range of hydrolases targeting both proteins and carbohydrates (Table 1). These could directly interfere with host immune effector molecules (e.g. antibodies, complement factor, acute phase proteins) and thereby supplement the immune gene regulating effect of other molecules.

## Conclusion

In the present study, we used adult wild-type zebrafish to establish an inflammation model using LPS and investigated modulation of immune genes expression after exposure to different concentrations of *C*. *osculatum* ES compounds. Inflammatory reactions were successfully induced in zebrafish not only by i.p. injection of LPS, but also by ES protein injection. The *C*. *osculatum* ES products exhibited in some cases a significant influence on expression of genes otherwise up-regulated by LPS exposure, but the composed nature of ES products can direct host reactions differently. Therefore, future work must isolate individual components in the ES mixture and precisely determine the pathways affected by individual proteins. The mixed responses described in this work may mimic the natural condition of host infection (mammalian or fish intermediate host) with the *C*. *osculatum* larvae; while parasite larvae penetrate into the host tissue and induce inflammatory immune responses, they also produce ES compounds with a potential to reduce inflammatory reactions. This may be interpreted as an evolutionary important ability, as this will minimize the host response towards the worm and thereby increase survival and subsequent reproduction.

## Supporting information

S1 Fig General gene expression levels. For each gene the ΔCq of all samples was calculated using the elongation factor 1α (*EF-1α*) as reference gene. The level of expression (2^-ΔCq^) was normalized to *IL-6* having the lowest expression level. Thus, *IL-6* is appearing with the mean of 1. High values indicate high expression levels.
(TIF)

S1 Table Primers and probes used for immune genes expressed in zebrafish. (DOCX)

S2 Table Genes expressed at low levels. A few genes were expressed at low levels, especially in the non- stimulated group (^C^). This will exclude using the -ΔΔCq method for assessing the fold regulation quantitatively. Thus, these genes were assessed qualitatively (regulation or not) rather than assessed quantitatively by recording the presence of valid Cq values (^a^) [23]. The non-parametric Mann-Whitney’s U-test (^b^) was used to test for differences between the stimulated groups against the non- stimulated group. From each group 10 fish were sampled (*P < 0.05; **P <0.01; ***P < 0.001; NS: no significant difference).
(DOCX)
