# Supplementary figures and images for: Immunomodulatory effects of excretory/secretory compounds from Contracaecum osculatum larvae in a zebrafish inflammation model
Source: PLoS One. 2017 Jul 24;12(7):e0181277. doi: 10.1371/journal.pone.0181277 (PMC5524353; doi:10.1371/journal.pone.0181277)

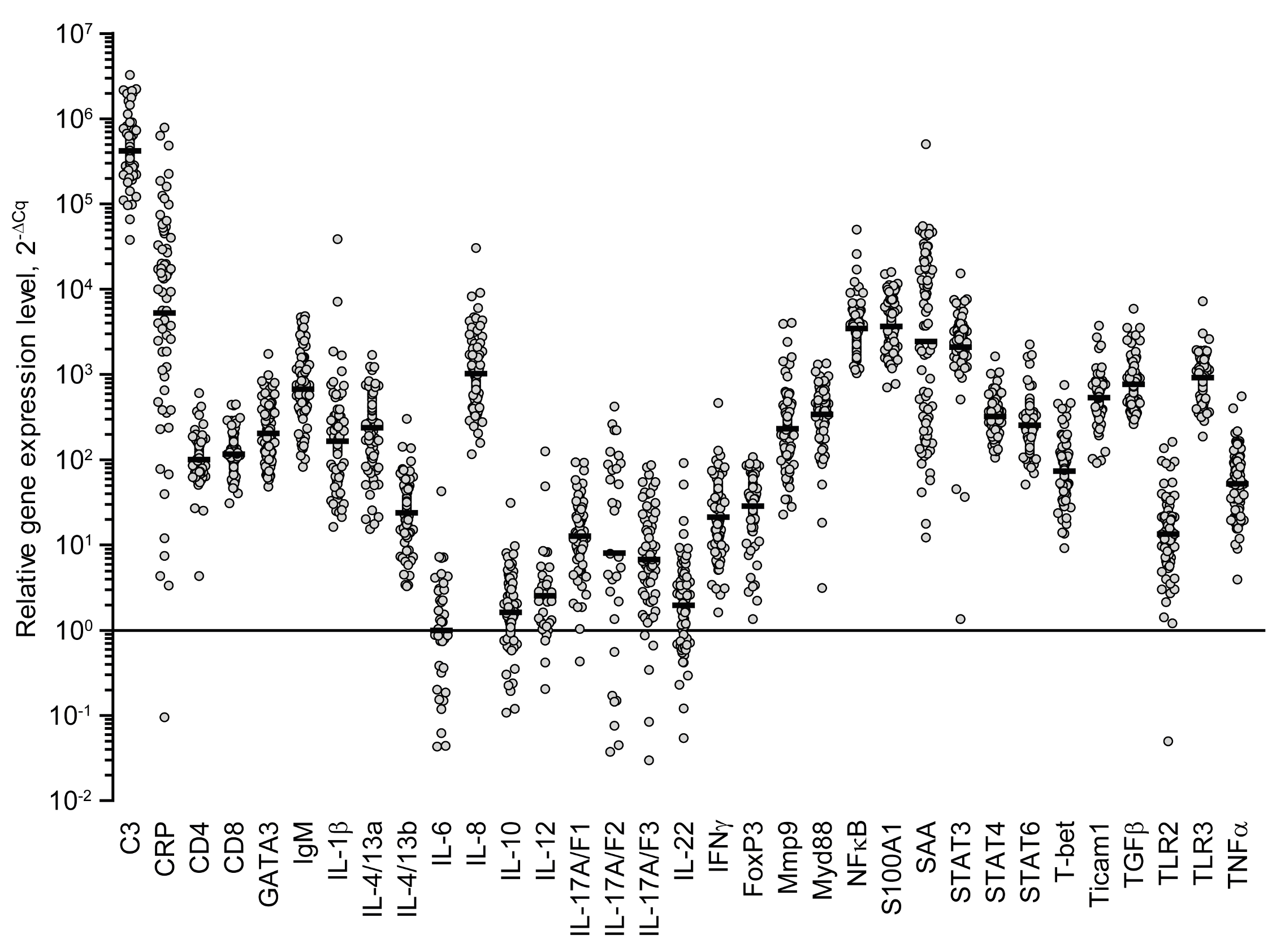

Supplement: S1 Fig — For each gene the ΔCq of all samples was calculated using the elongation factor 1α (EF-1α) as reference gene. The level of expression (2-ΔCq) was normalized to IL-6 having the lowest expression level. Thus, IL-6 is appearing with the mean of 1. High values indicate high expression levels. (TIF) [file pone.0181277.s001.tif]
