# Supplementary material for: Immunomodulatory effects of excretory/secretory compounds from Contracaecum osculatum larvae in a zebrafish inflammation model
Source: PLoS One. 2017 Jul 24;12(7):e0181277. doi: 10.1371/journal.pone.0181277 (PMC5524353; doi:10.1371/journal.pone.0181277)
Supplement: S1 Table — (DOCX) [file pone.0181277.s002.docx]

**S1 Table**

| **Genes** | **Forward primers** | **Reverse primers** | **Probes** | **Amplicon (bp)** | **Accession No.** |
| --- | --- | --- | --- | --- | --- |
| *ELF-1α* | GAACGACCCACCCATGGAGG | TGATGACCTGAGCGTTGAAG | GAACGACCCACCCATGGAGG | 158 | AY422992 |
| *RPL13* | TCCCAGCTGCTCTCAAGATT | ACTTCCAGCCAACTTCATGG | CACACGCAAATTTGCCCTGC | 87 | NM_212784 |
| *β-actin* | CCATCCTTCTTGGGTATGGA | ACAGGTCCTTACGGATGTCG | TGCGGTATCCACGAGACCACC | 91 | BC154531 |
| *IL-1β* | CGCTCCACATCTCGTACTCA | ATACGCGGTGCTGATAAACC | GAAGGAGACCGGCAGCTCCA | 166 | BC098597 |
| *IL-4/13A* | GTCAGGCTGAGGAGGAGATG | AGCAGCGTGACTCCTGATCT | CTGGCCTGTCCGGTGTCAAA | 155 | AB375404 |
| *IL-4/13B* | AACTCTCTGCCAAGCAGGAA | AAACGCTGCAGTTTCCAGTC | GAGACAGCTGAATGCTTATGCAGCA | 113 | AB375405 |
| *IL-6* | AGACCGCTGCCTGTCTAAAA | CAACTTCTCCAGCGTGATGA | TCCGCATGGACTCGCAAGAC | 293 | JN698962 |
| *IL-8* | GATCTGTCTGGACCCCTCTG | GGGCATTCATGGTTTTCTGT | CCATGGGTTAAGAAGATCATTGATAGG | 79 | XM_001342570 |
| *IL-10* | CTTGCCAAAATCCCTTTGAA | ATCAAGCTCCCCCATAGCTT | TGAAAAGATGAAGGAAAAGGGGG | 92 | BC163038 |
| *IL-12* | AGCAGGACTTGTTTGCTGGT | TCCACTGCGCTGAAGTTAGA | TAACTCGTCCTGCTCGGCCC | 145 | AB183001 |
| *IL-17 AF1* | GCTTTCTTATGGTGGCTTGC | GCCGGTATGAATGATCTGCT | ATCATTCGGTGCTGAGGGGG | 123 | AB195256 |
| *IL-17 AF2* | TCAATCTGAGGACGGAAAGG | GCTCCATCTCCTGTTTCAGC | CATCTTGCCCACTGGTGTGGA | 215 | NM_001020798 |
| *IL-17 AF3* | GGCTGCACATGTGTTTTACC | CAATGTGATTCGTTTTCAGGCT | CAGCGCCTATAGAAATAATCACTCCG | 250 | AB195260 |
| *IL-22* | GGATTACGCCAAAGGTGAAA | CGAGCACAGCAAAGCAATAA | CGACATCGAGGAACAACGGTG | 187 | NM_001020792 |
| *IL-23* | ATTACCGACCTGCCAGTGAC | TTCAGGAGGTGGCCAGTAAC | AAATGCCGAACAGATCGCCA | 212 | FN869917 |
| *IFNγ* | TATGGGCGATCAAGGAAAAC | CTTTAGCCTGCCGTCTCTTG | CGATCGTCCAGCGAAAGGCT | 129 | AB158361 |
| *TGFβ* | TGCGCAAGCTTTACATTGAC | AGGACCCCATGCAGTAGTTG | TGGATCCACAAGCCCAAGGG | 93 | AY178450 |
| *TNFα* | GCGCTTTTCTGAATCCTACG | AAGTGCTGTGGTCGTGTCTG | TGCACGCAGGAGCCTGAATC | 169 | AY427649 |
| *TLR2* | TGTCTCCCACCCTGAAACTC | GCCACTCTCCTATCCCAACA | CCTCCACGACCGATCAAGCC | 227 | AY388399 |
| *TLR3* | AAAGGGCTACGTTTGGTGTG | GTTGGTGGAGTTCAGCCATT | TCGCAAGAATTTTCGCCATTTTG | 119 | BC107955 |
| *Ticam1* | CAAGGATAGCAGGGTTGGAA | TTGTCGCACAAACTCTCCTG | TCGCAAGAATTTTCGCCATTTTG | 162 | BC134858 |
| *Myd88* | TGATGATCCACAGGGACTGA | GAGATGACCACCACCATCCT | CACATGCGTGTGGACCATCG | 222 | NM_212814 |
| *CD4* | CCAGACTGGAGCCAAGAGAC | CAGTGCAGCTCCACATCACT | CCTGCGCACAGTCAGGGAAA | 133 | EF601915 |
| *CD8* | TCGGAGGTTGTGGACTTTTC | TAATGGTGGGGACATCGTCT | CGTTCTGCTGATCGCAACCA | 88 | BC114236 |
| *IgM* | TGCAGTTCTGGTTCTGATGG | TGCACAAAATCGCTCAAATC | AATCACCCTCGGCTGCTTGG | 122 | AY643753 |
| *IgZ* | ATTGGATGTCTGGCCTCTGA | AATGCTGGGTGACGTTTTTC | TGCACAAAATCGCTCAAATC | 91 | AY643750 |
| *Mmp9* | ACAGGGAGACGCTCATTTTG | TGTTCCCTCAAACAGGAAGG | CGGTAATGCTGAGGGTGCAATGTGT | 124 | BC160656 |
| *FoxP3* | GACTTCTGCAACCTGCATCA | CGTATCATGGAGGCGTAGGT | GAGAGAGCAGCAACTGAAGCGC | 181 | FM881778 |
| *GATA3* | TACGTGTCCCGCTTAAAACC | TGAAGGGGCAATGAAGAAAG | GGCCTTCACTTTCGCCTGCT | 81 | BC162389 |
| *NFκB* | CAGGATTTGGACAAYGAGGT | CCAGTTCCCTCCAAGGTACA | TGAGCAAGCTGTGTGGGATTCT | 76 | BC122885 |
| *STAT3* | AGGGAGGAATCACATTCACG | GGCGACACTAGGATGTTGGT | CCAGATTCAGTCGGTTGAACCG | 160 | NM_131479 |
| *STAT4* | TATGAGAGCGATCCCATTCC | CCTGACCTTGGTGGTGAACT | TTGTGGTGGAGAAGCAGCCC | 174 | NM_001004510 |
| *STAT6* | TAAAGAGCTGGCGTCGAAAT | TCCCTGTTGTCCACATCAAA | GCCAGCTGGATAGAGGGGCA | 193 | BC162530 |
| *T-bet* | AAATCCAGGAGCATGGACAG | TGAGACTGGATGTGGGTTTG | TGGTTTTCCTATTGGAAGGCGG | 86 | AM942761 |
| *SAA* | CTTGCTGTGCTGGTGATGTT | CTTCCAATTGGCCTCTTTCA | CGCTGGAGGTGCAAAGGACA | 129 | BC081487 |
| *CRP* | TCGATAGGGAGGTCATCCTG | GCAGCAGGGTCTTTCTGACT | GATGCAGCGTTTTTCCGGCT | 163 | JF772178 |
| *C3* | TGCTGTTCTTCTCTCCTCAGC | WGAKGAACCCACTCTCAGCA | CTCACACTGTGTGACCCGCTAT | 88 | NM_131242 NM_131243 NM_001037236 |
