# Supplementary material for: Immunomodulatory effects of excretory/secretory compounds from Contracaecum osculatum larvae in a zebrafish inflammation model
Source: PLoS One. 2017 Jul 24;12(7):e0181277. doi: 10.1371/journal.pone.0181277 (PMC5524353; doi:10.1371/journal.pone.0181277)
Supplement: S2 Table — A few genes were expressed at low levels, especially in the non- stimulated group (C). This will exclude using the -ΔΔCq method for assessing the fold regulation quantitatively. Thus, these genes were assessed qualitatively (regulation or not) rather than assessed quantitatively by recording the presence of valid Cq values (a) [23]. The non-parametric Mann-Whitney’s U-test (b) was used to test for differences between the stimulated groups against the non- stimulated group. From each group 10 fish were sampled (*P < 0.05; **P <0.01; ***P < 0.001; NS: no significant difference). (DOCX) [file pone.0181277.s003.docx]

**S2 Table**

| **Gene** | **Treatment/**  **Concentration of ES (µg/mL)** | **^C^ PBS**  **0.00** | **PBS**  **50** | **PBS**  **500** | **PBS**  **1000** | **LPS**  **0.00** | **LPS**  **50** | **LPS**  **500** | **LPS**  **1000** |
| --- | --- | --- | --- | --- | --- | --- | --- | --- | --- |
| *IL-6* | ^a^ No. Valid Cq | 1/10 | 3/10 | 1/10 | 2/10 | 3/10 | 10/10 | 8/10 | 9/10 |
|  | ^b^ U-test |  | NS | NS | NS | NS | *** | ** | *** |
| *IL-12* | ^a^ No. Valid Cq | 2/10 | 2/10 | 2/10 | 4/10 | 7/10 | 6/10 | 5/10 | 7/10 |
|  | ^b^ U-test |  | NS | NS | NS | * | * | NS | * |
| *IL-17A/F2* | ^a^ No. Valid Cq | 0/10 | 2/10 | 3/10 | 6/10 | 4/10 | 6/10 | 4/10 | 5/10 |
|  | ^b^ U-test |  | NS | * | ** | * | ** | * | ** |
| *IL-17A/F3* | ^a^ No. Valid Cq | 2/10 | 9/10 | 6/10 | 8/10 | 8/10 | 10/10 | 10/10 | 10/10 |
|  | ^b^ U-test |  | ** | * | ** | ** | *** | *** | *** |
